# Evolutionary law and regulatory technology of roof migration on gob-side entry retaining

**DOI:** 10.1038/s41598-024-56108-z

**Published:** 2024-03-07

**Authors:** Li-Xin Zhang, Li Yi, Li Gang, Guang-Chao Liu, Ze-Hui Deng, Jia-Le Mi

**Affiliations:** School of Energy and Mining Engineering, Liaoning Technical University, Fuxin, 123032 Liaoning China

**Keywords:** Gob-side entry retaining, Evolutionary law of roof migration, Pressure relief by roof cutting, Civil engineering, Mineralogy

## Abstract

In order to study the evolutionary law of roof migration on Gob-Side Entry Retaining, this paper takes the gob-side entry retaining in the comprehensive mining face of the Ningtiaota coal mine as the engineering background, and analyzes the evolutionary law of the overlying rock layer on the roof at different locations during the roadway stay and the stress distribution around the roadway through numerical simulation software, which shows that there is a concentration of stress inside the Flexible formwork concrete wall, and therefore the maximum settlement of the roof on the side of Flexible formwork concrete wall is 35.35 mm, due to the existence of “arch-shaped” decompression area from the working face. Therefore, the maximum settlement of the roof slab on the side of flexible formwork concrete wall was 35.35 mm. Due to the existence of “arch-shaped” decompression area on the roof and floor of roadway, the settlement of the roof slab on both sides of the roadway gradually increased when it was from − 20 to 10 m away from the working face, and the central position had the following pattern of firstly decreasing and then gradually increasing, and then exceeding the top of the roadway. After decreasing and then gradually increasing, after 10 m ahead of the working face, the two sides of the roadway roof subsidence law and the central part of the roadway to maintain the same; the use of cutting the top of the flexible mold concrete wall support technology as a means of controlling the top of the roof along the empty roadway subsidence, the analysis shows that the roof after roof cutting of the amount of subsidence have been reduced, the maximum difference in the rate of change of the displacement is 0.011%, and the maximum difference in the amount of subsidence of 4.98 mm; through the field monitoring data analysis of the pressure of mining The peak value of the influence curve of the working face is located at 19 m of the working face, 9 m of the lagging working face and 19 m of the roadway outside the working face are less affected by the additional mining stress field, comparing the fracture brokenness of the roadway roof before and after the roof cutting, the fracture area in the uncut section is much larger than that in the section of the roof cutting.

## Introduction

As a result of large-scale mining, the amount of coal resources is decreasing, and the problems of high mining cost and low coal recovery rate caused by the traditional mining method of Remaining Coal Pillars and gob-side entry driving are becoming more and more prominent. Moreover, gob-side entry driving often causes coal pillar stress concentration, which triggers gob side entry peripheral rock deformation, impact pressure and coal (rock) and gas protrusion and other coal mine geological disasters. No coal pillar mining technology is a major innovation in coal mining method, which can not only reduce the mining cost, improve the resource recovery rate, alleviate the tension of mining and excavation succession, avoid the coal mine geological disasters produced by Remaining coal pillars, and solve the problem of gas accumulation in the upper corner of the working face, which is one of the main directions of the sustainable development of the coal industry.

As one of the key technologies of coal pillar mining^1–3^, non-pillar coal mining of the gob-side entry retaining improves the coal extraction rate, saves a large amount of high-quality coal resources, reduces the roadway digging rate, and accelerates the speed of mining and excavation succession, which is an important way to realize the safe and efficient mining of the mine^4^. However, gob-side entry need to be retained adjacent to the working face of the roadway as the current working face back to mining roadway use, the roadway by the mining and static and dynamic loads continue to perturb the impact^4–7^, the mine pressure appears intense, the roadway roof sinking compared to other roadways, therefore, mastering the evolutionary law of roof migration of gob-side entry retaining^8–10^ to maintain the stability of gob-side entry is particularly important for effective support^11^. At present, domestic and international scholars have carried out a lot of research on the stability of the peripheral rock of the roadway. Yang^12^ determined the degree of influence of different geological factors on the gob-side entry retaining based on the hierarchical analysis method; Wang^13^ investigated the distribution characteristics of the peripheral rock bias stress and the damage zone of the roadway during the mining process of the gob-side entry retaining. Lu Shiliang^14^ analyzed the fracture and collapse characteristics of the roof in the goaf in the gob-side entry and the force characteristics of the gob-side entry; Qin^15^ analyzed the stress distribution law on the gob-side entry retaining and made a clear explanation for the phenomenon of high-stress “nucleation” in the advancing process of the working face; Liu^16^ investigated the influence of the close coal seam roadway through numerical simulation and on-site monitoring. Liu^16^ investigated the roof damage mechanism of the gob-side entry retaining in the close coal seam through numerical simulation and on-site monitoring; Wu^17^ systematically analyzed the damage area, damage degree and range, and the dynamic evolution process of the rock on the gob-side entry retaining under four typical roof conditions in the mines from the peripheral rock plasticity zone; Xu^18^ adopted a particle flow model to study the fracture evolution of the rock in the process of the fracture of the overlying roof in the gob-side entry, and the stress and porosity; Gao Yongge^19^ used a particle flow model to study the fracture evolution of rock in the process of fracture of the overlying roof on the gob-side entry and the stress and porosity of rock. porosity; Gao Yongge^19^ monitored the degree of roof slab fragmentation and delamination of the roof slab in the primary and secondary mining respectively; because the gob-side entry retaining is located at the edge of the mining zone, the stability of the surrounding rock of the roadway has a close relationship with the roof activity law, the current roof control technology of the gob-side entry is based on the theory of masonry beams^20–22^, the theory of the cantilever beams^23–26^ and so on to establish the roof mechanical model and the stress distribution characteristics^21^, and the roof mechanical model^22^. and stress distribution characteristics^27^, and less research on the roof transport law at different locations of the roadway^28^, failing to provide strong data support for the maintenance of the stability^29–31^ of the surrounding rock of the gob-side entry^32–34^.

In this paper, with the background of Shaanxi Coal Group lemon tower coal mine comprehensive mining face along the empty stay roadway, through the use of numerical simulation software, the study of the roadway in the stay roadway during the different positions of the roof of the overlying rock layer transport law, the distribution of the stress around the roadway, compare the roof of the roadway roof of the overlying rock layer through the top of roof cutting and without roof cutting of the displacement rule of change, combined with the on-site monitoring data, to analyze the overrunning position and the position of the roadway of the lagging changes in the pressure data, the roof of the layer off the floor and the fissures, analyze the roadway along the empty overburden rock layer transport law and put forward the corresponding control technology.

## Method

**Figure 1:**
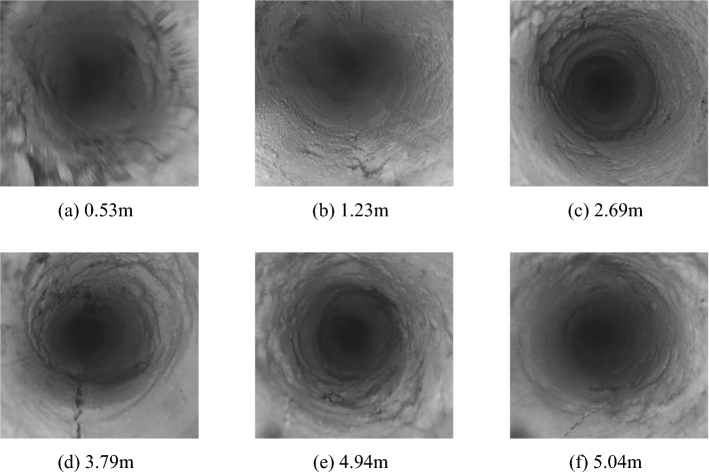
Borehole peephole.

Due to the Ningtiaota coal mine to retain the current working face roadway as the next working face using the gob-side entry retaining technology, and only use the thickness of 1 m flexible formwork concrete wall as the gob-side entry support, in the early stage of the gob-side entry retaining, the roadway stress concentration degree is large, the roadway overburden rock settlement is large, through the borehole peeping to analyze back to mining roadway deformation damage of the overburden rock layer as shown in Fig. 1.

As shown in Fig. 1, the rock wall was fractured at the orifice; a more obvious oblique longitudinal fissure with a length of about 0.15 m appeared at 1.23 m; the rock was fractured at 2.69 m; a clear longitudinal fissure with a length of about 0.3 m appeared at 3.79 m; the rock was fractured at 4.94 m with a fracture with a length of about 0.1 m; at 5.04 m the rock appeared with a number of fissures with a length of about 0.1 m; at 5.04 m the rock strata appeared to have multiple more obvious fissures and a longitudinal fissure with a length of about 0.09 m. Before cutting the roof, there were many fissures in the overlying rock layer along the roof of the open roadway, and the condition of the roof was poor, which was not favorable for the maintenance of the gob-side entry.In order to better maintain the stability of the gob-side entry, it is very important to grasp the roadway roof evolutionary law, this paper through the numerical simulation and on-site inspection to study the roof evolutionary law, first of all, introduce the numerical simulation model establishment process.

**Figure 2:**
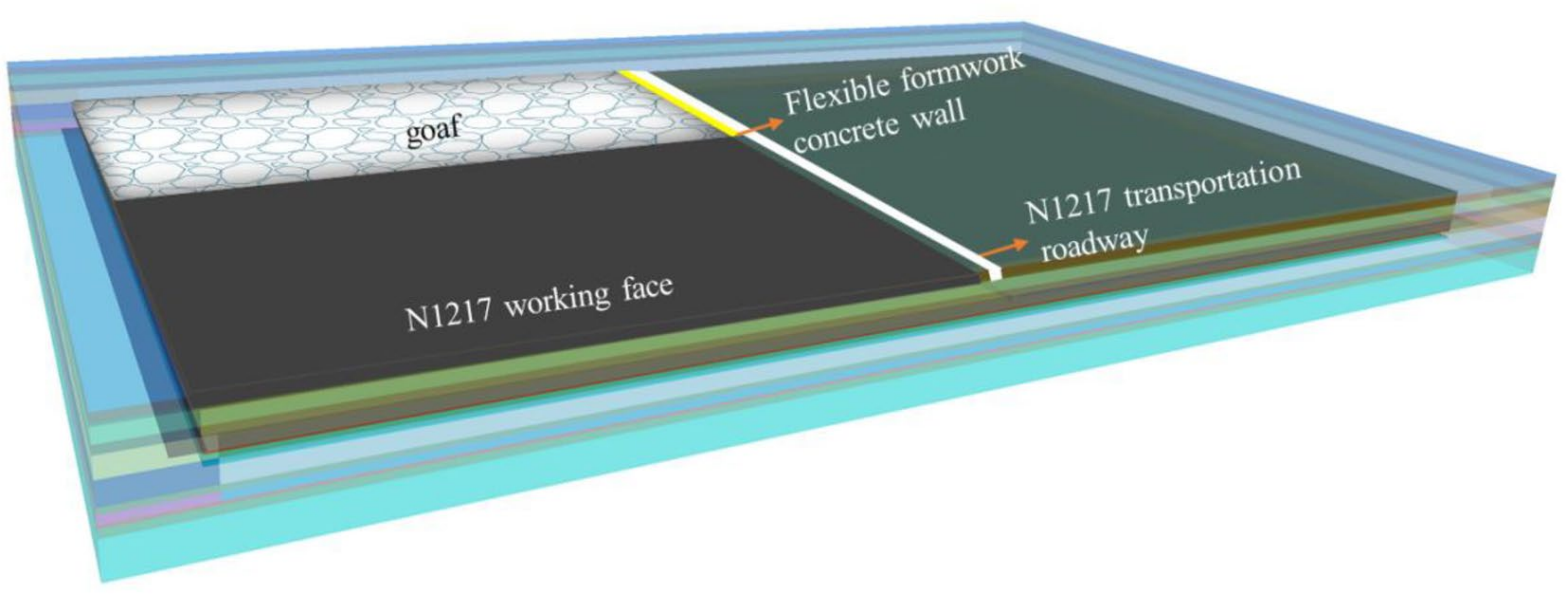
Numerical simulation model.

Taking the engineering geological conditions of the N1217 working face of Ningtiaota coal mine as the background, FLAC3D simulation software is used to simulate the evolutionary law of roof migration during the period of gob-side entry retaining, and the numerical model shown in Fig. 2 is established, after analyzing and researching, reasonable simplification is carried out for the geological conditions, and the model is reduced as much as possible after ensuring that the model can truly reflect the actual situation, so as to ensure the stability and accuracy of the model calculations. The final scale of the model is 600 m × 500 m × 60 m; in order to eliminate the boundary effect, 50 m width is left to protect the coal pillar; the roadway is left along the bottom of the coal seam, with the height of 3.5 m and the width of 3.8 m; the working face is mined back for 150 m, and as the working face advances, the Flexible formwork concrete wall support is carried out on the side of the mining hollow area, which is constructed by Flexible formwork concrete wall. As the working face advances, Flexible formwork concrete wall support is constructed at the side of the hollow area, and the N1217 transportation roadway is retained as the return roadway of the next working face through the Flexible formwork concrete wall support; the boundaries around the model and the lower boundaries are set as the fixed displacement boundaries.

Since the initial stress field is the basis for analyzing the redistribution of stress in the surrounding rock in the mining space, in order to carry out the engineering simulation simulation more realistically, it is necessary to ensure the reliability of the initial ground stress field that already exists before the simulated mining. According to the actual geological conditions of the site, the vertical initial stress of the simulated coal seam is calculated according to the weight of its overlying rock layer, and the average volume force of the overlying rock layer is 25 KN/m^3^; As the depth of the coal seam in Ningtiaota coal mine is 213 m, a vertical load of 5.15 MPa is applied to the upper part of the model to simulate the self-weight of the overlying loose layer, and the gravity acceleration of the model is set to be 10 m/s^2^, and the initial lateral pressure coefficient is 1.2.

Numerical simulation model calculations use Mohr–Coulomb damage criterion, from Mohr–Coulomb principal model to determine the required rock mechanical parameters, according to the engineering research data and adjacent mine geological data, to determine the physical and mechanical parameters of the rock layer and overburden rock, due to the use of C30 concrete in the flexible formwork wall with the tensile anchors as the support material along the hollow stay, so the flexible formwork concrete wall is still using Mohr–Coulomb principal model, through the laboratory test and field monitoring data on the flexible formwork concrete wall parameter assignment is shown in Table 1. Since C30 concrete with tensile anchors is used as the supporting material along the open channel, the flexible formwork concrete wall still adopts the Mohr–Coulomb principal model, and the parameters of the flexible formwork concrete wall are assigned with parameters through the laboratory test and on-site monitoring data, and the parameters are set as shown in Table 1^35^.

**Table 1:** Parameters of rock mechanical.

Lithology	Bulk (GPa)	Shear (GPa)	Density (kg m^−3^)	Tension (MPa)	Cohesion (MPa)	Friction (°)
Medium-grained sandstone	2.86	2.12	2450	1.5	4.2	30
Fine-grained sandstone	6.4	2.9	2410	8.1	4.1	36
Silt stone	2.57	2.31	2200	2.5	4.5	30
Sandy mudstone	2.2	1.6	2360	1.9	2.1	29
Coal	0.79	0.71	1450	0.5	0.9	27
Concrete wall	5.2	3.5	2450	3.5	5.5	45

In this simulation, the N1217 working face needs to consider the compaction process of its internal coal rock body, and the double yield constitutive model (Doubleyield) is suitable for simulating the geotechnical materials that will produce irrecoverable compression deformation and shear yielding, and the use of the double yield constitutive model to fill the goaf can more realistically reflect the change rule of the stress around the goaf. Therefore, this simulation chooses the double yield constitutive model to simulate the N1217 working face mining goaf, and the model parameters are set as shown in Table 2.

**Table 2:** Parameters of collapsed zone.

Density (kg m^−3^)	Bulk (GPa)	Shear (GPa)	Dilation (°)	Friction (°)	Cohesion (MPa)
1806	14.36	10.33	8	1	0.001

**Figure 3:**
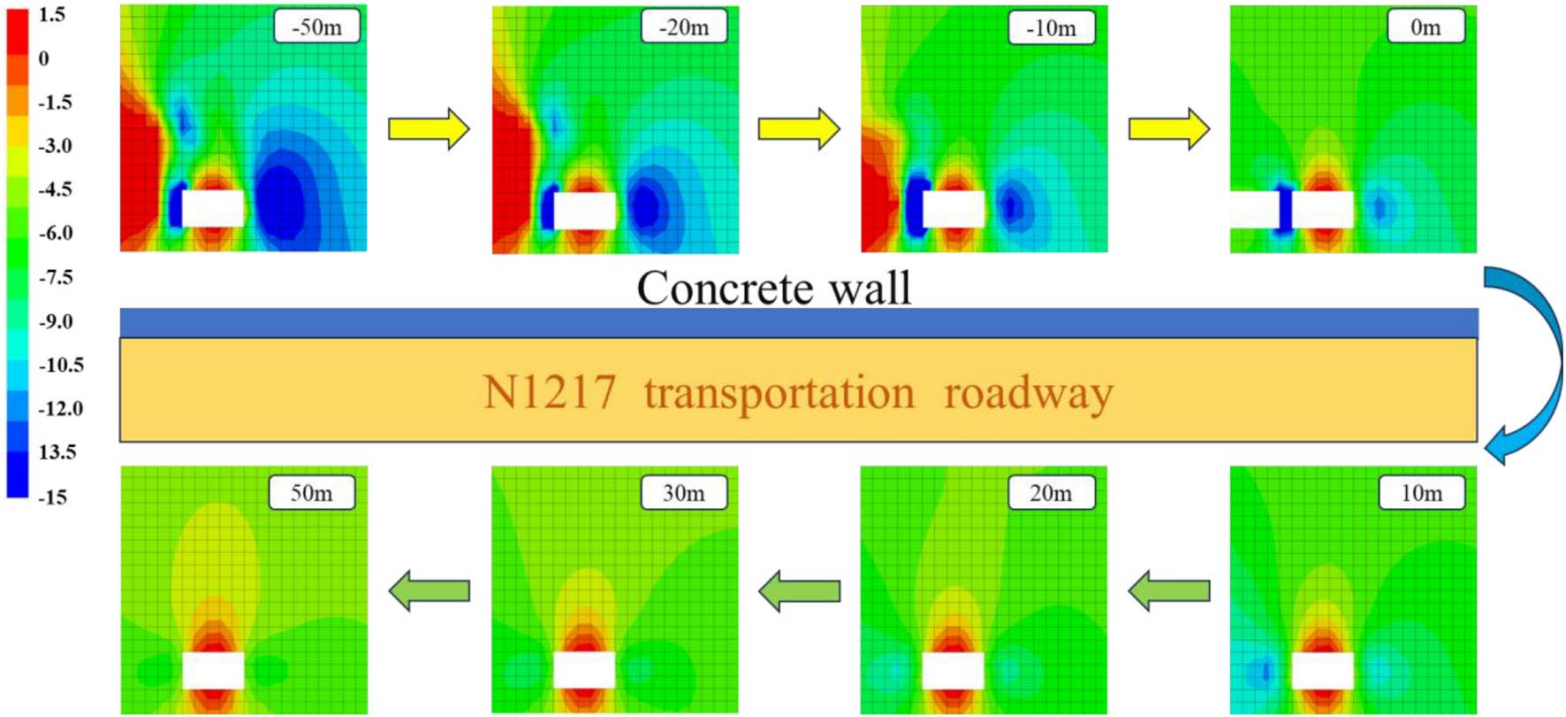
Vertical stress evolution law of gob-side entry.

As shown in Fig. 3, the vertical stress distribution cloud diagram of the lagging end and overrunning section of the N1217 transportation roadway is intercepted, and it can be seen from the diagram that, during the workface mining, the lagging section adopts the double yielding model to simulate the collapse of the cavitation zone, and there is a tensile stress in the interior of the cavitation zone, and the maximum value of the stress is at the direct top of the workface, with the stress value of 1.07 MPa. Due to the influence of working face mining and roadway excavation, stress concentration occurs inside the flexible formwork concrete wall, and the stress is in the form of tensile stress, and the maximum stress is located in the middle of the positive gang of the roadway, and the maximum value reaches 14.92 MPa, and due to the excavation of the roadway, the natural balanced arch occurs, and the roof and floor of roadway are released, and the roof and floor appear in the shape of “arch”. The maximum tensile stress in the area is 0.97 MPa, and the stress concentration is transferred to the gang part. Under the influence of mining, a wide range of tensile stress is generated at the working face, so a wide range of compressive stress area is generated at the side of the negative gang, and the maximum stress concentration is in the horizontal direction of the roadway, with the concentration value of 13.55 MPa, and a part of the compressive stress concentration area is generated in the overlaying rock layer of the roadway, with the value of 12.43 MPa. 12.43 MPa; up to the end of the working face, the stress distribution pattern is basically the same, but due to the − 10 m position from the working face is in the stress reduction area, and the − 20 m and − 50 m positions from the working face are in the original rock stress area, so the stress value from the − 10 m position of the working face is smaller compared to other areas, and the stress value at the negative gang side and the top of the roadway is reduced by 5.57 MPa; when at the end of the working face, the stress value around the roadway is reduced by 5.57 MPa, and the stress value in the negative gang side and the top of the roadway is reduced by 5.57 MPa. At the end of the working face, the stress around the roadway is basically symmetrically distributed, the roof and floor of roadway appear to be “arched” pressure relief area, and the two gangs of the roadway appear to be concentrated in compressive stress, and at the 10 m position from the working face, due to the position is in the area of influence of the working face in the overrun, the degree of stress concentration in the positive gang is greater than that of stress concentration in the negative gang, and the maximum stress in the positive gang side is 11.92 MPa, and in the negative gang side, the maximum stress is 11.92 MPa. 11.92 MPa, the maximum stress value of negative gang side is 9.12 MPa, the stress difference between the two gangs is 2.8 MPa, when farther away from the working face, the difference of stress concentration gradually decreases, when 20 m away from the working face, the stress difference between the two gangs is reduced to 1.47 MPa, 30 m away from the working face, the stress concentration and stress value of the two sides of the roadway basically remain the same, with the further away from the working face, the “arch-shaped” decompression zone of the roof and floor of the roadway also expanded, and basically covered the whole roof at 20 m.

**Figure 4:**
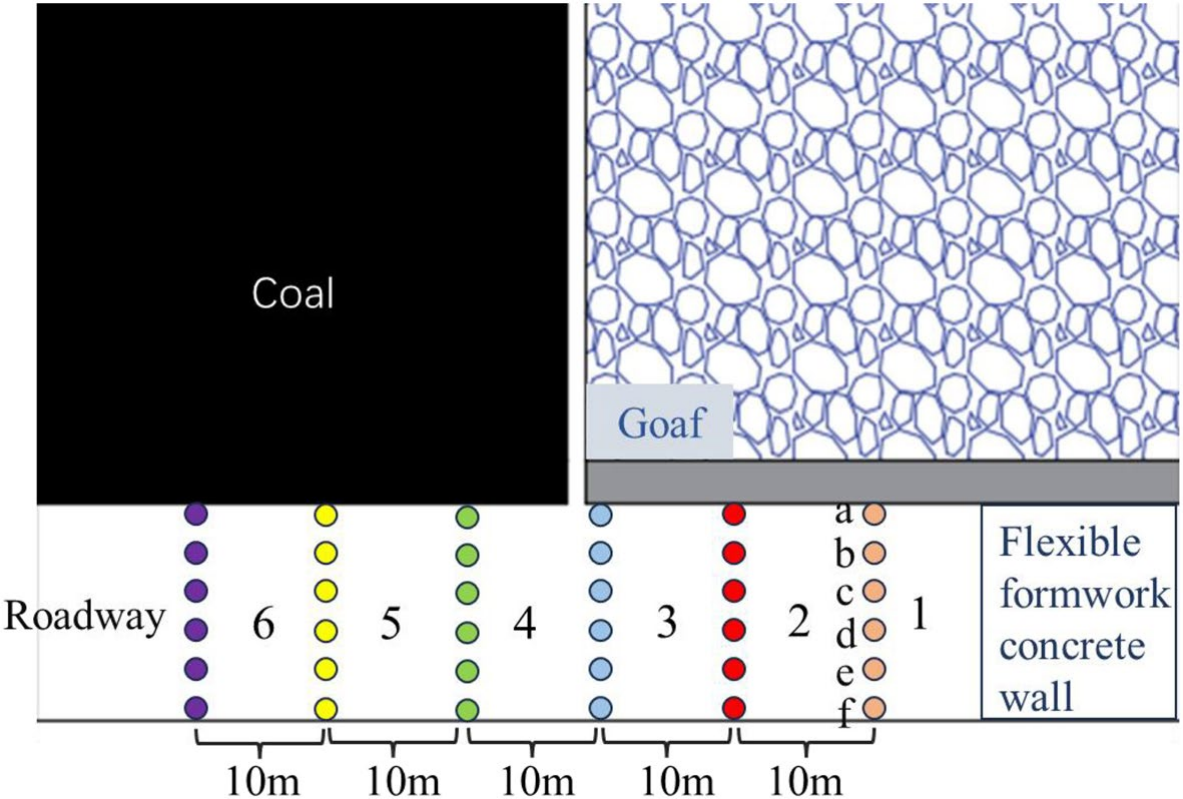
Layout of roadway measurement points.

**Figure 5:**
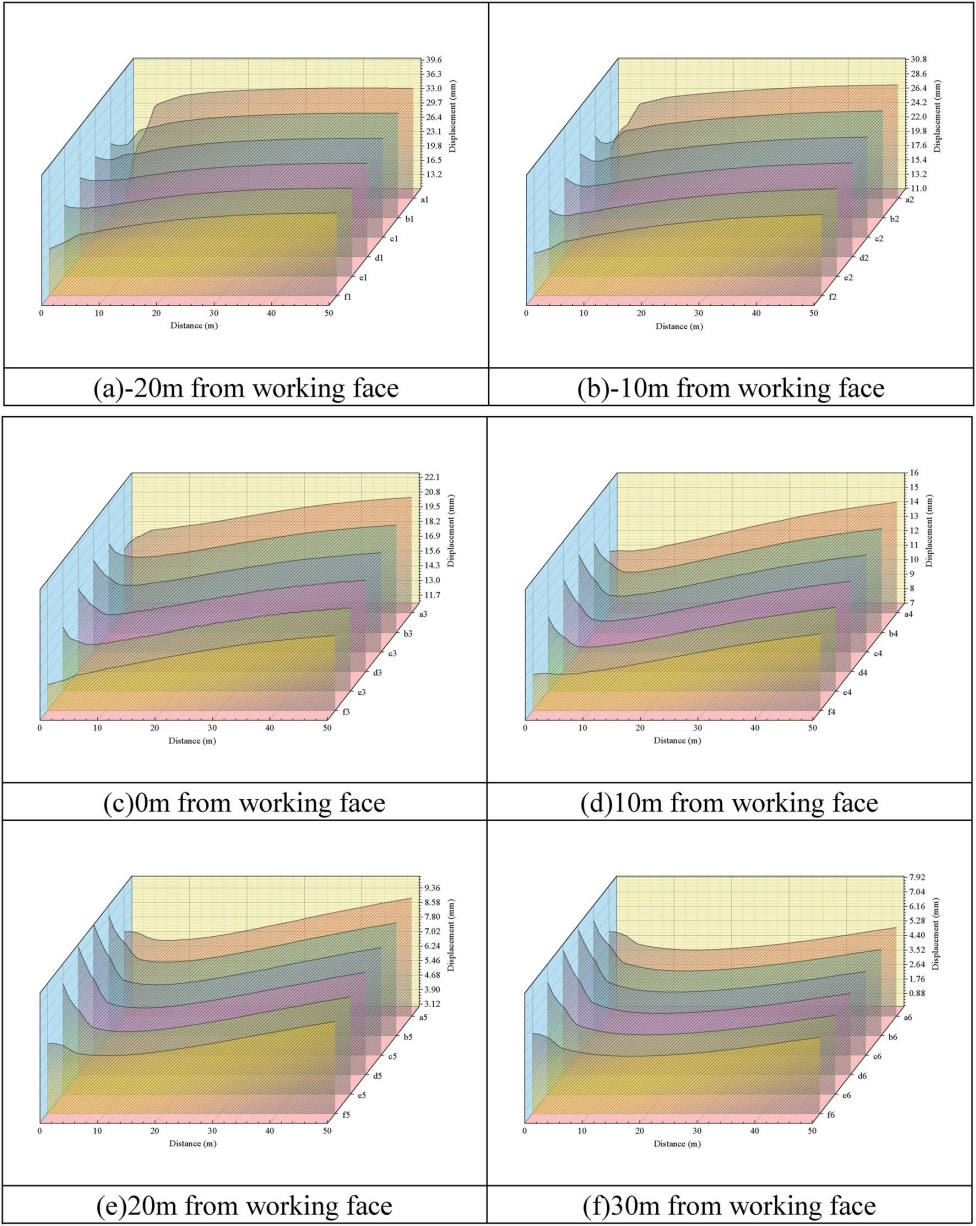
Change curve of roadway roof subsidence.

By arranging measurement points at different locations in the gob-side entry, evolutionary law of roadway roof migration of the overreach section, the working face and the lagging section is compared and analyzed, and five measurement points are arranged on the same horizontal line to analyze and compare the roof transport law of the roadway roof at different locations and the change of the sinking amount, and the results of the specific analysis of each measurement point are shown in Fig. 5, and the arrangement of the measurement points is shown in Fig. 4.

As shown in Fig. 5, at − 20 m from the working face, the roof at position a1 sinks the most, with a sinking of 35.35 mm within 50 m, and a displacement change rate of 0.707%, the roof at position f1 sinks the least, with a sinking of 29.05 mm within 50 m, and a displacement change rate of 0.058%, and the roof at position b1, c1, d1, and e1 has basically the same pattern of sinking, which can be seen in Fig. 3. It can be seen that, due to the influence of mining, and the stress redistribution caused by the excavation of the roadway, the vertical stress of the roof within 5 m at b1, c1, d1 and e1 is in the depressurization zone, and the amount of roof subsidence decreases with the distance from the ground surface within the range of 5 m, and the roof enters into the state of compressive stress after leaving the tensile stress range of 5 m, and the amount of compressive stress increases with the decrease in distance from the ground surface. The position of a1 is the top of Flexible formwork concrete wall, which is the stress concentration area with the largest compressive stress, so the roof sinking law at a1 is gradually and slowly increasing, and at f1, due to the solid coal on one side of the mining side, the stress on the roof is transferred to the solid coal range during the roadway excavation, and the roof is only subject to the compressive stress at f1, so the phenomenon of decreasing and then increasing does not occur. In − 10 m from the working face, the maximum subsidence of the roof is still at a1, and the subsidence is 28.41 mm within 50 m, the displacement change rate is 0.057%, the change rule of subsidence is basically the same as that of − 20 m from the working face, which is still gradually increasing at a1 and f1, and decreasing and then increasing at b1, c1, d1, and e1, which are in the area of stress reduction, therefore, the change value of subsidence is relatively low compared with that of − 20 m from the working face. Therefore, the change value of subsidence is small compared with that of − 20 m from the working face; on the side of the working face, the rule of change of the roof subsidence is basically the same as that of the lagging section, and the largest change of subsidence is still at a1, with a subsidence of 21.51 mm, and the change rate of displacement in the range of 50 m is 0.043%; when it is 10 m away from the working face, the change of displacement of a1, f1 within 5 m tends to be smooth, and it starts to appear linearly after 5 m. When 10 m from the working face, the displacement of a1 and f1 in the range of 5 m tends to be gentle, and beyond the range of 5 m, there is a linear increment, b1, c1, d1 and e1 in the range of 5 m, the magnitude of reduction increases, and the maximum reduction is from 13.66 to 10.53 mm, and the maximum subsidence is 14.63 mm in the range of 10 m from the working face, and the rate of change of the displacement is 0.057%; in the range of 20 m from the working face, the pressure release zone is reduced due to the arching of the roof of the tunnel, which is the most important factor for the displacement. At 20 m from the working face, due to the expansion of the “arched” pressure relief zone, the five measured lines of the roadway roof show the trend of decreasing and then increasing, the decreasing amount at a1 and f1 is small, and the difference of the change amount in 5 m is 2.99 mm compared with the other positions, and the change value of the overall subsidence amount is basically the same, and the average amount of subsidence in the range of 50 m is 8.54 mm, and the maximum amount of the roof subsidence is still 8.54 mm at 20 m from the working face, the maximum amount of the roof subsidence at a1 is 14.63 mm, and the rate of change of displacement is 0.057%. a1, the roof subsidence decreases from 5 to 16.7 m, after 16.7 m, the roof subsidence gradually increases, the roof subsidence within 50 m is 5.47 mm, the displacement change rate is 0.011%.

## Result

**Figure 6:**
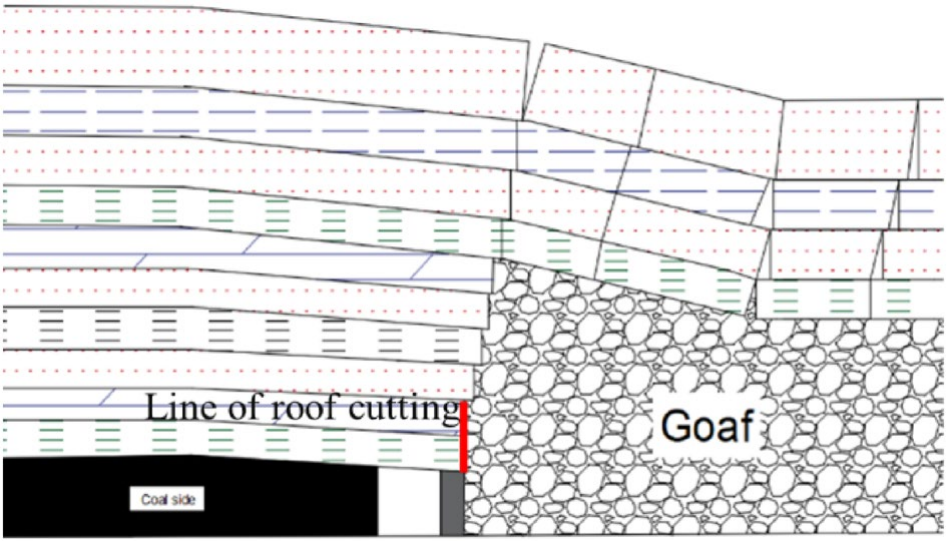
Schematic of the roof cutting position.

Due to the degree of mine pressure manifestation of the gob-side entry is more intense than other mining conditions, through a large number of scholars research^36^, Through the roof plate directional slit, cut off part of the roof plate of the mine Pressure transfer, forming a “short-armed roof beam”.the overlying roof of the mining roadway to relief pressure by roof cutting, the basic top of the fracture in gob-side entry to transfer the location of the fracture to the side of the goaf, can effectively reduce the deformation damage of the roadway rock, optimize the rock stress of the gob-side entry with the roof structure, shorten the time of the gob-side entry by the impact of the mining movement, and is conducive to the rapid stabilization of rock structure in the gob-side entry. Therefore, we adopt the roof cutting technology as the rock damage and roof settlement control technology of the gob-side entry, and simulate the effect of roof cutting by setting up the contact surface of the roof along the flexible formwork concrete wall side of the gob-side entry in Flac 3D, and the location of the roof cutting line is shown in Fig. 6. The roof settlement trend is compared between without roof cutting and roof cutting on the gob-side entry.

**Figure 7:**
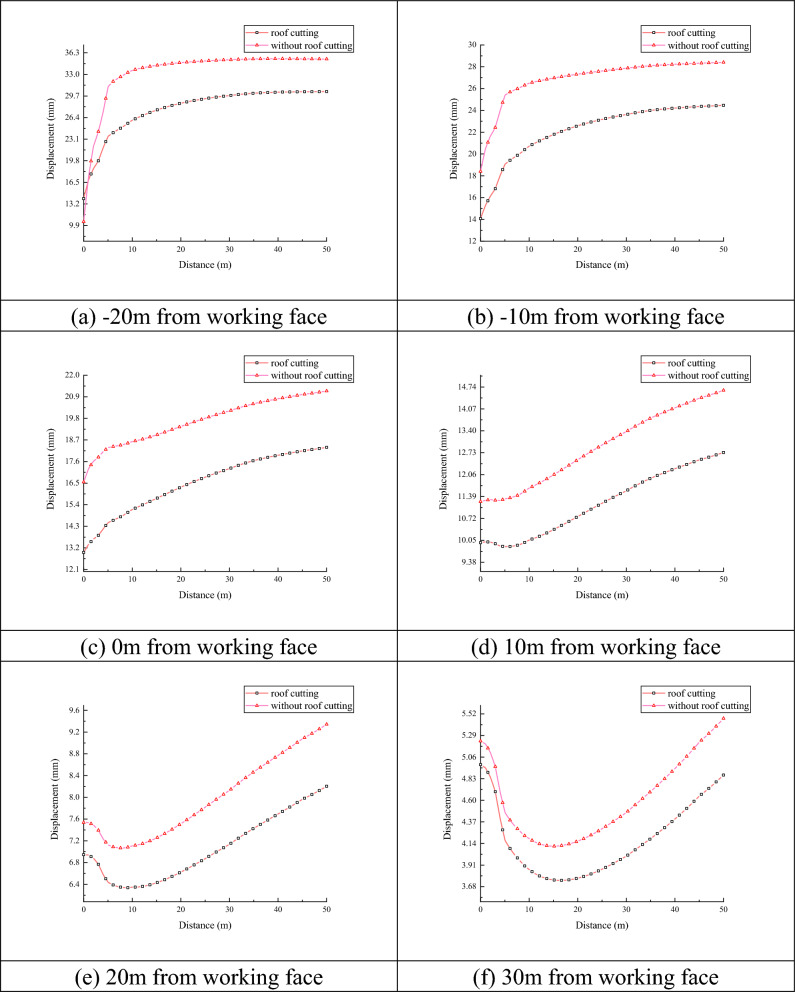
Comparison of roof subsidence before and after roof cutting.

As shown in Fig. 7, the location of the five measuring points with the largest displacement change in the horizontal direction of the roadway, i.e., a1, is selected to analyze the displacement change curves of the over-advanced section and the lagging section, as shown in Fig. 7a, when − 20 m away from the working face, the maximum displacement in the range of 50 m before the top-cutting was 35.35 mm, and the rate of change of the displacement was 0.071%, and the maximum displacement in the range of 50 m after the top-cutting was 30.37 mm, and the rate of change of displacement was 0.060%, and after the top-cutting, the trend of roof settlement gradually slowed down, which represents the weakening of the dynamic pressure of the roadway. The displacement change rate is 0.060%, after cutting the top, the trend of roof subsidence is gradually slow, that is to say, it represents that the degree of dynamic pressure of the roadway is weakened; as shown in Fig. 7b, when it is − 10 m from the working face, the amount of subsidence in the range of 50 m before cutting the top is 28.41 mm, and the displacement change rate is 0.057%,, and the maximum displacement in the range of 50 m after cutting the top is 30.37 mm, and the displacement change rate is 0.049%, the settlement pattern of the roof is basically the same as that of the uncut roof, and the displacements are greatly decreased; as shown in Fig. 7c, when it is on one side of the working face, the roof sinking amount is 21.51 mm before roof cutting, and the rate of change of the displacement in the range of 50 m is 0.043%, and the maximum displacement in the range of 50 m after roof cutting is 18.32 mm, and the rate of change of the displacement is 0.036%; as shown in Fig. 7d As shown in Fig. 7d, when 10 m away from the working face, the roof subsidence within 50 m before roof cutting is 14.63 mm, the displacement change rate is 0.057%, the roof subsidence within 50 m after roof cutting is 12.73 mm, the displacement change rate is 0.025%, and the change of the roof subsidence changes from gradual incremental to first decrease within 5 m, and then gradually increase; as shown in Fig. 7e, when 20 m away from the working face, the maximum displacement is 18.32 mm, the displacement change rate is 0.036%; as shown in Fig. 7e, when 20 m away from the working face, the maximum displacement is 18.32 mm, the displacement change rate is 0.036%. As shown in Fig. 7e, when the working face is 20 m away, the roof settlement law is basically the same as that at 10 m away from the working face, the roof subsidence within 50 m before roof cutting is 9.34 mm, and the displacement rate of change is 0.019%, and the roof subsidence within 50 m after roof cutting is 8.21 mm, and the displacement rate of change is 0.016%; as shown in Fig. 7f, the range of the roof subsidence decreasing is increased when it is 30 m away from the working face, the roof subsidence decreasing to 16.21 mm, and the roof subsidence decreasing to 16.21 mm, and the roof subsidence decreasing to 16.21 mm. As shown in Fig. 7f, when 30 m away from the working face, the roof subsidence decreases to 16.7 m and then gradually increases, the roof subsidence in the range of 50 m before roof cutting is 5.47 mm, and the rate of change of displacement is 0.011%, and the roof subsidence in the range of 50 m after roof cutting is 4.86 mm, and the rate of change of displacement is 0.009%.

Through the above curve analysis, the maximum amount of roof subsidence of the roadway is compared with the maximum amount of roof subsidence after cutting the top, and the maximum amount of subsidence is − 20 m from the working face, the difference in the rate of change of displacement is 0.011%, and the maximum difference in the amount of subsidence is 4.98 mm. Through numerical simulation, roof cutting to relief pressure is effective in maintaining the stability of gob-side entry and controlling the subsidence of the roof.

## Discussion

**Figure 8:**
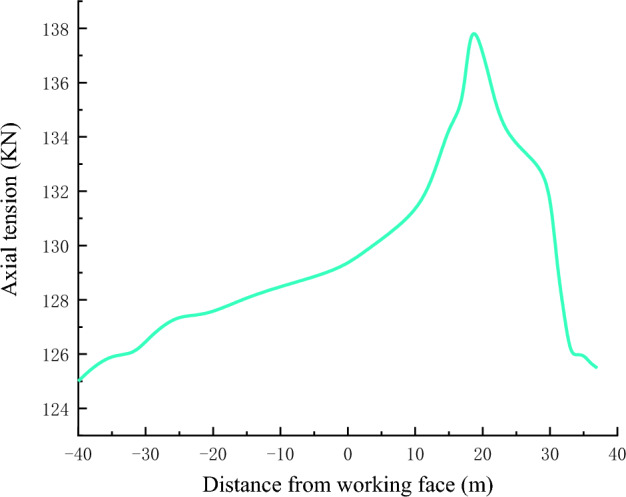
Anchor cable axial force.

Through the anchor cable dynamometer to monitor the axial tension of the anchor cable along the roof of the empty roadway, as shown in Fig. 8, when 19 m from the working face, the axial tension of the anchor cable on the roof of the roadway is the largest, with the maximum value of 137.92 KN. It can be seen that 19 m is the peak of the influence curve of the overrunning abutment pressure in the working face, and the anchor cable force decreases greatly after leaving the influence of the range of the abutment pressure. After lagging the working face by 9 m, with the gradual collapse and compaction of the roof on the mining side, the anchor cable force decreases rapidly again, and then the decreasing trend gradually flattens out.

**Figure 9:**
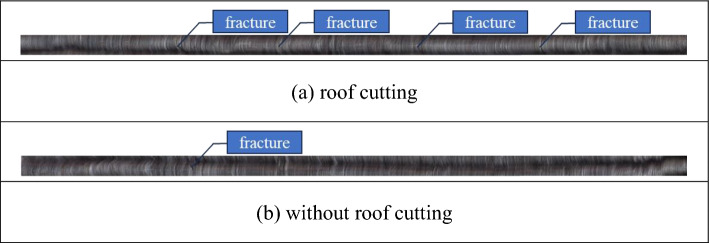
Comparison of roof cracks before and after roof cutting.

The drill hole peeping before and after roof cutting and generating the drill hole histogram of the overlying rock layer were carried out at 20 m of the overrunning working face respectively. As shown in Fig. 9, due to the drastic reduction of the roof sinking after roof cutting, the stress distribution around the roadway has also decreased, and four fissures appeared on the roof of the back-mining roadway without roof cutting to remove the pressure, and one fissure appeared on the roof after roof cutting to remove the pressure and the overburden rock layer was more stable.

## Conclusion

This paper adopts numerical simulation analysis and on-site mine pressure monitoring means, analyzes the roadway in different positions during the stay on the roof of the overlying rock layer transport law, the roadway around the distribution of stress, comparing the roadway roof of the overlying rock layer after roof cutting of the displacement change rule without roof cutting, and ultimately concluded that using the way of roof cutting for the roof regulation and control, the effect is significant, this paper draws the specific conclusions are as follows:

1. At − 20 m from the working face, the roof has the largest amount of settlement, and the largest displacement is the roof on the side of flexible formwork concrete wall, and the amount of subsidence is 35.35 mm, and the amount of subsidence on both sides of the roof of the roadway is gradually increasing when it is from the − 20 to 10 m from the working face, and the subsidence law of the central position is first decreasing and then gradually increasing, and the roadway is in line with that of the central part after exceeding the working face by 10 m. After 10 m ahead of the working face, the subsidence law of both sides of the roof is consistent with that of the center.
2. Due to the influence of working face mining and roadway excavation, stress concentration occurs inside the Flexible formwork concrete wall, and the stress is in the form of tensile stress, and the maximum value of stress is located in the middle of the roadway gang; due to the excavation of the roadway, the roof and floor of the roadway get the stress release, and then the roof and floor appear in the form of "arched" decompression area, and the roof and floor are in the shape of "arch". "With the farther away from the working face, the "arch-shaped" pressure relief area of the roof and floor of the roadway also expands, and basically covers the whole roof at 20 m.
3. After the roof cutting, the amount of roof subsidence was reduced, and the largest change in the amount of subsidence was at − 20 m from the working face, with the difference in the rate of change of displacement of 0.011% and the largest difference in the amount of subsidence of 4.98 mm. roof cutting to relief pressure had a better effect on maintaining the stability of the gob-side entry retaining and controlling the roof subsidence.
4. Through the analysis of on-site monitoring data of mining pressure, the peak value of the influence curve of the working face is located at the 19 m of the working face, and the roadway outside the 9 m of the lagging working face and the 19 m of the working face is less affected by the additional mining stress field; comparing the fracture of the roadway roof before and after the top-cutting, the fracture area in the uncut section is larger than that in the roof cutting section.

## Data Availability

The raw data supporting the conclusion of this article will be made available by the authors, without undue reservation. Li Yi (lhf_lhf2022@126.com) should be contacted if someone wants to request the data from this study.
